# Supplementary material for: MetaRibo-Seq measures translation in microbiomes
Source: Nat Commun. 2020 Jun 29;11:3268. doi: 10.1038/s41467-020-17081-z (PMC7324362; doi:10.1038/s41467-020-17081-z)
Supplement: Supplementary file 10 — Supplementary Data 7 [file 41467_2020_17081_MOESM10_ESM.zip › File2/Confidence_High_Taxonomy/16234_out.krona.html]

Javascript must be enabled to view this page.

members
magnitude
magnitudeUnassigned
count
unassigned
taxon
rank

16234\_out

2

2
superkingdom
2

phylum
976
2

2
200643
class

171549
order
2

family
171552
2

1
1283313
genus

712469
species

SRS063272\_contig\_number\_contig-100\_672.672
1

1
genus
838


SRS016133\_contig\_number\_14088
1
1433844
species
